# Linking surveillance and clinical data for evaluating trends in bloodstream infection rates in neonatal units in England

**DOI:** 10.1371/journal.pone.0226040

**Published:** 2019-12-12

**Authors:** Caroline Fraser, Berit Muller-Pebody, Ruth Blackburn, Jim Gray, Sam J. Oddie, Ruth E. Gilbert, Katie Harron

**Affiliations:** 1 UCL Great Ormond Street Institute of Child Health, University College London, London, United Kingdom; 2 National Infection Service, Public Health England, London, United Kingdom; 3 Institute of Health Informatics, University College London, London, United Kingdom; 4 Microbiology, Birmingham Women’s & Children’s Hospitals, Birmingham, United Kingdom; 5 Bradford Neonatology, Bradford Royal Infirmary, Bradford, United Kingdom; 6 Centre for Reviews and Dissemination, University of York, York, United Kingdom; University of Illinois College of Medicine, UNITED STATES

## Abstract

**Objective:**

To evaluate variation in trends in bloodstream infection (BSI) rates in neonatal units (NNUs) in England according to the data sources and linkage methods used.

**Methods:**

We used deterministic and probabilistic methods to link clinical records from 112 NNUs in the National Neonatal Research Database (NNRD) to national laboratory infection surveillance data from Public Health England. We calculated the proportion of babies in NNRD (aged <1 year and admitted between 2010–2017) with a BSI caused by clearly pathogenic organisms between two days after admission and two days after discharge. We used Poisson regression to determine trends in the proportion of babies with BSI based on i) deterministic and probabilistic linkage of NNRD and surveillance data (primary measure), ii) deterministic linkage of NNRD-surveillance data, iii) NNRD records alone, and iv) linked NNRD-surveillance data augmented with clinical records of laboratory-confirmed BSI in NNRD.

**Results:**

Using deterministic and probabilistic linkage, 5,629 of 349,740 babies admitted to a NNU in NNRD linked with 6,660 BSI episodes accounting for 38% of 17,388 BSI records aged <1 year in surveillance data. The proportion of babies with BSI due to clearly pathogenic organisms during their NNU admission was 1.0% using deterministic plus probabilistic linkage (primary measure), compared to 1.0% using deterministic linkage alone, 0.6% using NNRD records alone, and 1.2% using linkage augmented with clinical records of BSI in NNRD. Equivalent proportions for babies born before 32 weeks of gestation were 5.0%, 4.8%, 2.9% and 5.9%. The proportion of babies who linked to a BSI decreased by 7.5% each year (95% confidence interval [CI]: -14.3%, -0.1%) using deterministic and probabilistic linkage but was stable using clinical records of BSI or deterministic linkage alone.

**Conclusion:**

Linkage that combines BSI records from national laboratory surveillance and clinical NNU data sources, and use of probabilistic methods, substantially improved ascertainment of BSI and estimates of BSI trends over time, compared with single data sources.

## Introduction

Babies admitted to neonatal units (NNUs) have among the highest rates of health-care acquired bloodstream infection (BSI) of all patient groups.[1, 2] There is evidence that these high rates can be reduced by multifaceted interventions to improve asepsis, however adoption of such interventions varies by NNU.[3–5] Accurate measurement of trends over time in BSI is important to monitor effective implementation of improved asepsis and to identify NNUs and patient groups where practice is suboptimal.[6] Risk adjustment is essential for valid comparison of rates of BSI between units because of variation in susceptibility to infection according to gestational age at birth, number of invasive procedures, and duration of intravenous nutrition and hospital stay.[7, 8]

The primary source for monitoring BSI in England is the national laboratory surveillance system, coordinated centrally by Public Health England, which receives regular reports of BSI diagnosed by laboratories serving NHS patients. The surveillance dataset contains information on age and laboratory but no clinical information or whether a baby was admitted to NNU. The National Neonatal Research Database (NNRD) is derived from a clinical record system universally used for all babies admitted to a NNU and captures clinical information on BSI, but this is entered by clinicians as part of daily practice and is incomplete. Linkage of these national laboratory surveillance and clinical NNU databases is therefore required to accurately estimate risk-adjusted trends in BSI across England. However, improvements in data quality over time, changes in data collection methodology, and inconsistent reporting by laboratories, could all potentially bias observed trends in BSI.

This is the first linkage study involving national laboratory infection surveillance and NNRD clinical records in England. First, we evaluate linkage rates through comparison to clinical records of BSI in NNRD. Second, we show how linkage success changes over time with improved data quality. Third, we determine the added value of probabilistic linkage methods compared with deterministic linkage, when identifiers are recorded imperfectly.

## Methods

### Data sources

Clinical data from NNUs in England are captured by the BadgerNet electronic patient record system and routinely transferred to the Neonatal Data Analysis Unit at Imperial College London for research uses (the National Neonatal Research Database NNRD)[9] We restricted our analyses to data from NNUs that provide intensive and high dependency care (termed NICUs) and local neonatal units (LNUs) providing short term intensive care and high dependency care. We excluded data for low intensity care special care baby units.[10] Data captured in NNRD includes clinical characteristics, daily care procedures, and clinician-entered records of laboratory-confirmed blood or cerebrospinal fluid (CSF) infection that we refer to as a clinical record of BSI. BSI events are incomplete in NNRD, but this is improving.[11, 12]

The Neonatal Data Analysis Unit sought consent from a designated neonatologist at 122 NNUs that provided intensive or high dependency care in England to use data from their unit for the study. Forty four (96% of 46) neonatal intensive care units (NICUs) and 68 (89% of 76) local neonatal units (LNUs) consented. The remaining ten did not respond to three requests to participate in the study. We defined the study population as babies aged <1 year admitted to the 112 NNUs between January 2010 and June 2017. We received data from the NNRD in two waves: babies admitted between January 2010 and December 2015 and babies admitted between January 2015 and June 2017. This resulted in missing data (including discharge dates) for babies admitted in 2015 and discharged in 2016. We also missed data on babies who were in NNU in January 2010 but admitted previously. We therefore excluded data from January 2010 to February 2010 and September 2015 to February 2016.

Public Health England operates a national laboratory infection surveillance system to which microbiology laboratories serving NHS patients in England voluntarily submit reports on positive cultures. [13] Surveillance data contain sample type, date, age, organism(s) isolated and laboratory, but no clinical details and no information on whether or not a baby was admitted to a NNU. To capture any BSI episodes that might be associated with babies within our NNU study population, we extracted all positive blood or CSF cultures for infants less than 1 year old with a sample date between December 2009 and August 2017. We did not expect all of these records to link to NNRD, since some would belong to babies who were admitted to different wards (e.g. Paediatric Intensive Care), some would correspond to babies in NNUs who were not included in our sample, and some would correspond to admissions before or after our study period. We use the term BSI for organisms isolated from blood or CSF. We included CSF isolates within the definition of BSI because such isolates will have been haematologically seeded into the central nervous system implying a past bacteraemia. Repeat samples of the same organism from the same baby within 14 days of the first sample were classified as one episode. Only the first sample from each episode was included for linkage.

Reporting practices changed during the study period due to an update to the surveillance system. Between 2010 and 2014, laboratories were required to report only clinically significant results. Between 2014 and 2016, laboratories began reporting BSI to a new surveillance system. The new surveillance system allowed laboratories to report all results automatically with the option to deselect results that were not clinically significant. This system change resulted in a step increase from 2014 to 2016 in the number of BSI reported that were previously not considered clinically significant, for example skin organisms that may have been considered contaminants. We therefore included only BSI caused by clearly pathogenic organisms that we expect to have been reported consistently during the study period. We defined clearly pathogenic organisms as organisms which if cultured from blood or CSF would be indicative of a BSI without the need for repeat cultures or clinical signs (see list of included and excluded organisms in S1 Appendix). We excluded other organisms (n = 18,862) and organisms it was not plausible to culture from blood or CSF which we believed were errors (n = 275). We also excluded mixed cultures defined as cultures of multiple organisms from the same sample type, on the same day from the same source laboratory (n = 364). In addition to the system level changes, reporting by individual laboratories varied over time, due to staff changes or technical issues. We therefore manually inspected monthly data for each laboratory to identify reporting gaps, and excluded 356 of 8830 (4%) laboratory months. The corresponding NNU months were also excluded from analysis, to avoid overestimating the number of admitted babies who could have linked with a surveillance record.

We plotted the rates of missing patient identifiers (NHS number, date of birth, postcode prefix, postcode suffix and sex) over time for data from NNRD and the national laboratory infection surveillance data. Patient identifiers were cleaned separately before linkage and invalid identifiers were set to missing (see S2 Appendix for details).

### Linkage

We first used deterministic linkage to link babies in NNRD with BSI records in the surveillance data. For the deterministic linkage, we linked records where NHS number was the same in each dataset. Linkage was one to many, where a BSI could only link to one baby but babies could link to multiple BSI (e.g. if a baby had multiple BSI events during an admission). We discarded links with a sample date more than seven days before admission or more than 14 days after discharge to exclude BSI related to admissions outside NNU or to NNUs not included in our data. Our aim was to create a linked dataset that could support a range of analyses (e.g. BSI per baby/admission/bed-days), and so we used a simple definition of BSI for demonstration purposes (any BSI occurring between 7 days before admission and 14 days after discharge). We included days before and after the admission to allow for errors in dates but the time at risk can be restricted to identify BSI related to the neonatal admission in specific future analyses using the data.

For records that could not be linked deterministically, we used probabilistic linkage based on the remaining common identifiers in each dataset (date of birth, postcode prefix, postcode suffix, sex and hospital/laboratory). In probabilistic linkage, match weights are created that represent the likelihood that two records belong to the same subject, according to the similarity of a set of identifiers.[14] Full details of the probabilistic linkage methods and match weight calculations are given in S3 Appendix. In brief, we first used ‘blocking’ to restrict our comparisons to records that agreed on at least one of date of birth, postcode prefix or postcode suffix.[15] Next, in order to calculate the probabilistic match weights, we used the set of deterministic links (where NHS number agreed) as the reference standard for a true match.[16] This allowed us to estimate the probability that identifiers agreed, disagreed or were missing in either dataset, given records were a true match (m-probabilities). We estimated the probability of identifier agreement for records that were true non-matches by comparing identifiers in records that disagreed on NHS number (u-probabilities). Weights for each identifier were summed across each comparison pair. To determine whether records should be classified as links or non-links, we chose two thresholds. Record pairs with a weight above the upper threshold were considered to be links; record pairs with weights below the lower threshold were considered to be non-links. The thresholds were chosen based on inspection of a plot of the frequency of summed match weights (Fig A in S3 Appendix). Comparison pairs with a match weight between these two thresholds were reviewed manually by one author (CF) following a set of rules agreed by all authors (see S3 Appendix for details).

### Linkage rates

We derived the proportion of babies in NNRD that linked to at least one BSI in the surveillance data, using deterministic linkage and deterministic plus probabilistic methods. We conducted subgroup analyses for babies born before 32 weeks gestation, since these babies have a high risk of BSI and we expected linkage rates to be higher in this group.[17] We then derived the proportion of BSI records for babies <1 years recorded in national laboratory surveillance data that linked with a baby in NNRD. We also report rates during NNU admission, which are more clinically relevant, by restricting BSI to those with a sample date between two days after NNU admission and two days after NNU discharge.

### Linkage quality

To evaluate the quality of linkage, we estimated linkage rates for records we expected to link. First, we calculated the proportion of BSI record from considered babies aged <28 days in surveillance data that linked to a baby in NNRD. These babies were most likely to have been admitted to an NNU and therefore more likely to link to NNRD. Second, we calculated linkage rates for the subset of babies with a clinical record of BSI in NNRD, as we expected that all of these babies should have a link in the surveillance data. We estimated linkage rate (sample date between 7 days before admission and 14 days after discharge) and rate of BSI during admission (sample date between two days after admission to two days after discharge) for babies with a clinical record of BSI in NNRD.

We examined comparison pairs below the lower threshold with high levels of missing identifiers, to determine whether any of these could potentially be missed links. We considered BSI records as potential missed links if they only paired with babies with a weight below the lower threshold and were in pairs with multiple missing identifiers which if were present and in agreement could have been links as potential missed links (e.g. agree on date of birth but missing all other identifiers). We report the percentage of BSI records in the surveillance data for which true link status could not be determined due to high levels of missing identifiers. We plotted the frequency of these pairs over time, excluding any pairs that included BSI linked to another baby, to determine how missing identifiers may have affected linkage trends.

Following probabilistic linkage, we further inspected unlinked records in NNRD for babies aged <28 days with a clinical record of BSI in NNRD, as we believed these records should link to a BSI. For these babies, we searched the surveillance data for any BSI reported to the corresponding lab (determined by mapping NNU and laboratory pairings for records that linked) in the same month and reviewed the potential links (full detail are given in S3 Appendix).

To identify potential sources of bias (i.e. where particular groups of babies may have been less likely to link), we compared characteristics of i) babies with a clinical record of BSI in NNRD who linked with a surveillance data record indicating a clearly pathogenic organism ii) babies with a clinical record of BSI caused by clearly pathogenic organisms in NNRD who did not link with a surveillance data record, iii) babies without a clinical record of BSI caused by clearly pathogenic organisms in NNRD who linked with a surveillance data record, and iv) any babies in NNRD who linked with a surveillance data record, augmented with any unlinked babies with a clinical record of BSI caused by clearly pathogenic organisms in NNRD, i.e. “any record of BSI”. We used chi-squared tests to determine whether the distribution of age at BSI, gestational age at birth, year of sample and organism were significantly different (p<0.05) in unlinked babies with a clinical record of BSI or linked babies without a clinical record of BSI compared to babies with a clinical record of BSI and a link to the surveillance data. In this comparison we only included babies with a BSI caused by clearly pathogenic organisms in either dataset and restricted all analyses to BSI during NNU admission (between 2 days after admission and 2 days after discharge) to ensure we were comparing similar BSI in either dataset.

To assess differences in linkage success using deterministic compared to probabilistic over time, we compared the proportion of all links that were identified using deterministic linkage in July 2010-June 2011 and July 2016-June 2017. These periods had the lowest and highest quality of patient identifiers, respectively.

### BSI trends

We plotted the proportion of babies admitted to a NICU or LNU in NNRD who linked to a BSI per month (7 days before admission to 14 days after discharge), using deterministic linkage alone and BSI identified using deterministic plus probabilistic linkage. We then augmented these estimates by adding in clinical records of BSI in NNRD, for babies without a linked surveillance data record. For reference, we also show trends of BSI captured using only clinical records of BSI in NNRD. We used Poisson regression models to calculate rate ratios (RRs) for monthly change in the proportion of babies with BSI for each of the above groups. We did not include any risk-adjustment and considered a trend significant if the 95% confidence intervals (CI) for the annual change in rate did not include zero.

### Ethics

The NNRD have approval from the National Research Ethics Service (10/H0803/151) and the Confidentiality Advisory Group of the Health Research Authority to collect patient identifiable information without explicit patient consent (ECC-05(f)/2010). Public Health England has permission to use laboratory infection surveillance data without patient consent under Section 251 of the NHS Act 2006. Specific permissions for linkage using identifiable data between NNRD and the laboratory surveillance system was given by PHE in accordance with regulation 3 of The Health Service (Control of Patient Information) Regulations 2002.

## Results

### Data sources

We extracted 17,388 BSI episodes recorded in the national laboratory infection surveillance system for infants from 16^th^ December 2009 to 14^th^ August 2017. The NNRD extract comprised 349,740 babies (299,616 (86%) singletons and 50,124 (14%) babies from multiple births) admitted to the 112 NNUs in the study between 1^st^ March 2010 and 30^th^ June 2017.

**Fig 1:**
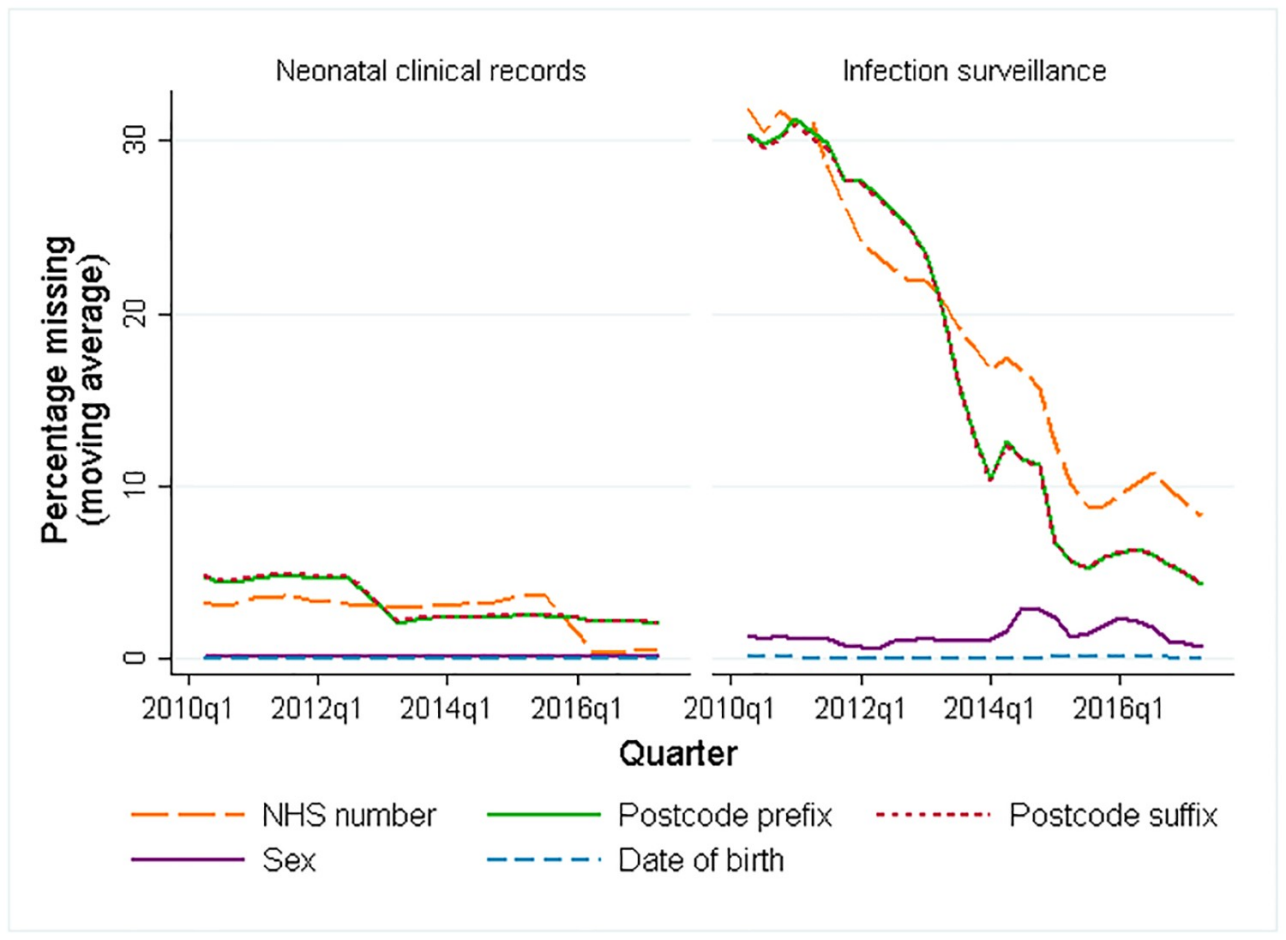
The rate of missing patient identifiers over time for 112 NNUs included in NNRD and national laboratory surveillance data. Moving average based on three quarters.

The completeness of postcode prefix, postcode suffix and NHS number improved over time in both datasets (Fig 1). All BSI recorded in the surveillance data had a completed date of birth and laboratory (since the data were extracted based on these variables). All NNRD clinical records included the reporting hospital.

### Linkage rates

**Fig 2:**
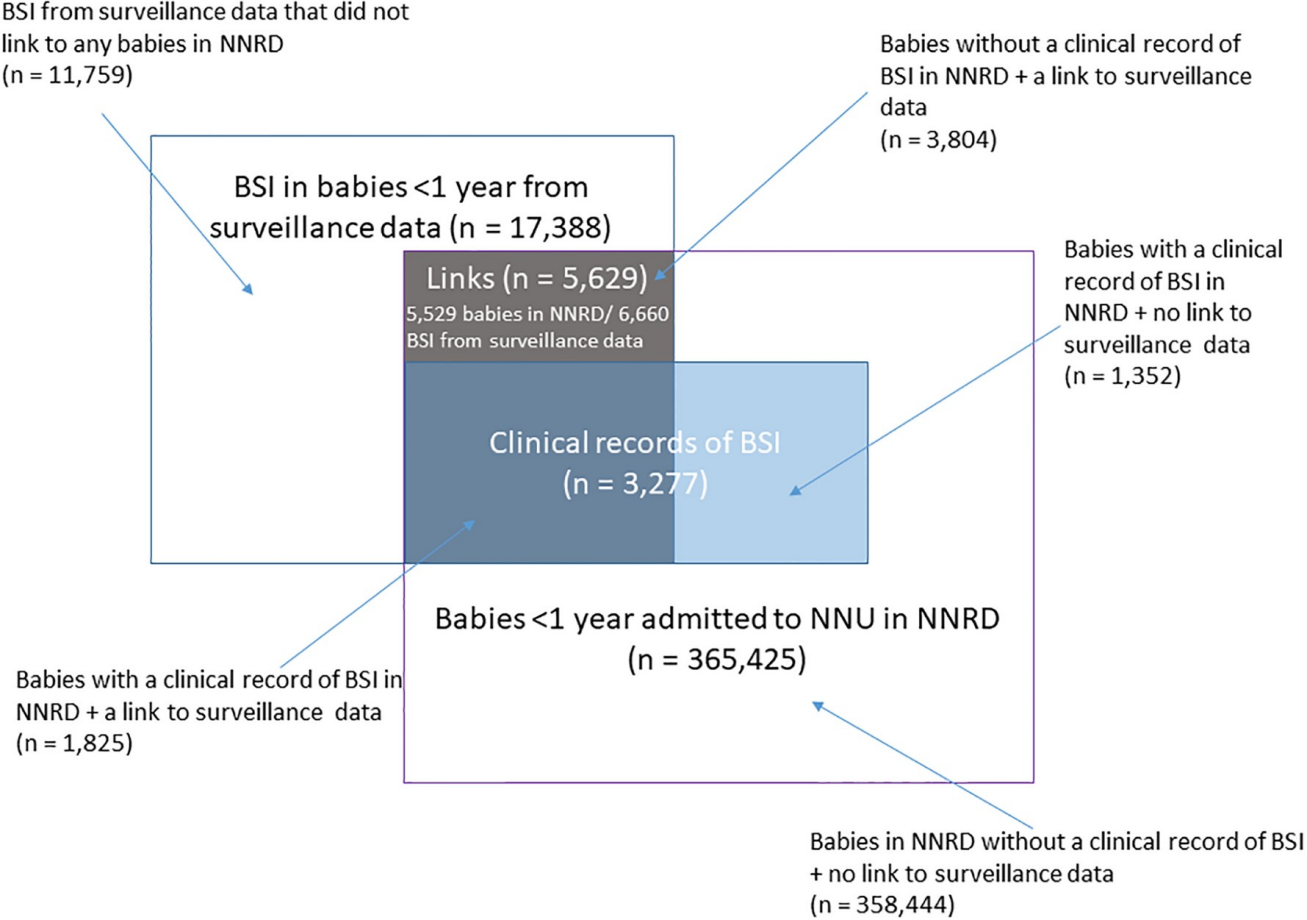
Diagram showing linked and unlinked records from surveillance data and NNRD (not proportional).

Using deterministic and probabilistic linkage together (primary measure), we linked 5,629 babies in NNRD (1.6% of 349,740 babies admitted to a NNU in NNRD) with 6,660 BSI (38% of 17,388 BSI records for babies aged <1 year in the surveillance data) (Fig 2). Full details of linkage including a flow diagram of the number of babies and BSI at each step are given in S1 Fig.

The proportion of babies with BSI using the primary measure was 1.6% (5,629 / 349,740) compared to 1.4% (5,011/349,740) using deterministic alone, 0.9% (3,277 / 349,740) using only clinical records of BSI in NNRD, and 2.0% (7,081 / 349,740) when augmenting the linked records with additional clinical records of BSI in NNRD that had not linked with the surveillance data. The equivalent rates when restricting to babies born before 32 weeks gestation in NNRD (i.e. those more likely to have a BSI) were 6.1% (3,112 / 50,921), 5.7% (2,916 / 50,921), 3.8% (1,914 / 50,921), and 7.6% (3,889 / 50,921). The average number of BSI episodes per baby with BSI was 1.18 (6,660 BSI in 5,629 babies) for all babies and 1.23 (3,838 BSI in 3,112 babies) for babies born before 32 weeks gestation.

The BSI rates decreased substantially when restricting the analyses to BSI with a sample date between two days after admission and two days after discharge (i.e. BSI associated with a NNU admission). The proportion of babies with BSI during neonatal admissions was 1.0% (3,148/302,543) using the primary measure, 1.0% (2,880/302,543) using deterministic alone, 0.6% (1,734 /302,543) using only clinical records of BSI in NNRD and 1.2% (3,729/302,543) when augmenting the linked records with additional clinical records of BSI in NNRD that had not linked with the surveillance data. The equivalent rates when restricting to babies born before 32 weeks gestation in NNRD were 5.0% (2,496 / 49,597), 4.8% (2,364 / 49,597), 2.9% (1,436 / 49,597), and 5.9% (2,951 / 49,597).

### Linkage quality

More than half (55%; 4,881/8,895) of BSI records (pathogens) for babies aged <28 days in the surveillance data linked to a baby in NNRD. The recording of BSI in NNRD increased over time (168 babies with BSI in 2010 compared to 392 in 2016). For babies with clinical records of BSI in NNRD (i.e. those we expected to link), 56% (1,825/3,277) were linked with BSI records from surveillance data (sample date between seven days before admission and 14 days after discharge). However, of the 6,660 linked BSI records, 4,473 (67%) linked with babies in NNRD that did not have a clinical record of BSI. For babies with clinical records of BSI in NNRD 65% (1,127/1,734) linked with BSI records from surveillance data during their admission to NNU (sample date between two days after admission and 2 days after discharge). Early onset BSI accounted for 42% (1,363/3,277) of clinical records of BSI in NNRD and were excluded from our estimate of clinical records of BSI during NNU (as were any other BSI which occurred within two days of NNU admission).

We could not determine link status (i.e. whether there was a link or not) for 1,681/17,388 (10%) BSI records in the surveillance data, due to high levels of missing identifiers. We could not determine whether these were babies that were admitted to paediatric services outside the NNU (and therefore should not have linked with NNRD), or whether there should have been a link that we were unable to capture due to identifier quality. This number changed over time: as identifier quality improved, the proportion of BSI records from surveillance data for which link status could not be determined declined (S2 Fig).

Overall, the characteristics of babies with a clinical record of BSI in NNRD differed from those who linked to a BSI record during NNU admission (between two days after admission to two days after discharge) (Table 1). Clinical records of BSI in NNRD caused by some pathogens (notably *S*. *aureus*) were less likely to link to surveillance data than other organisms (Table 1). Furthermore, the babies in the NNRD with a clinical record of BSI who did not link to a BSI record in the surveillance data tended to be younger at the time of BSI and were more likely to be born at term. Babies with a clinical record of BSI were less likely to link in earlier years.

**Table 1:** Characteristics of babies in 112 units recorded in NNRD with BSI caused by clearly pathogenic organisms during NNU admission from linkage to national infection surveillance data and/or clinical records of BSI.

	i) Babies with a clinical record of BSI in NNRD + a link to surveillance data (likely true matches)	ii) Babies with a clinical record of BSI in NNRD + no link to surveillance data (potential missed matches)	iii) Babies without a clinical record of BSI in NNRD + a link to surveillance data (BSI not recorded in NNRD)	iv) Any record of BSI ^a^: All babies with a clinical record of BSI in NNRD +/- a link to surveillance data
	Primary measure with (iii)		Primary measure with (i)	(sum of i) + ii) + iii))
**Total** (% of babies with any record of BSI)	1,046	688	1,995	3,729
**Age at BSI** ^b^				
2–6 days	138 (13%)	116 (18%)	306 (15%)	560 (15%)
7–13 days	259 (25%)	163 (25%)	478 (24%)	900 (24%)
14–20 days	183 (18%)	108 (17%)	275 (14%)	566 (15%)
21–27 days	121 (12%)	54 (8%)	20 (10%)	381 (10%)
28–365 days	345 (33%)	204 (32%)	730 (37%)	1,279 (35%)
**p-value from chi-squared test**		0.034	0.016	
**Gestation** ^b^				
<26	390 (27%)	212 (31%)	633 (32%)	1,235 (33%)
26 to <28	238 (23%)	152 (22%)	406 (20%)	796 (21%)
28 to <32	270 (26%)	174 (25%)	476 (24%)	920 (25%)
32 to <37	102 (10%)	73 (11%)	274 (14%)	449 (12%)
37+	46 (4%)	72 (11%)	206 (10%)	324 (9%)
**p-value from chi-squared test**		<0.001	<0.001	
**Year of sample**				
2010	44 (4%)	52 (8%)	295 (15%)	390 (11%)
2011	119 (11%)	109 (17%)	313 (16%)	541 (15%)
2012	166 (16%)	132 (20%)	250 (13%)	548 (15%)
2013	194 (19%)	113 (18%)	182 (9%)	489 (13%)
2014	201 (19%)	123 (19%)	200 (10%)	524 (14%)
2015	140 (13%)	69 (11%)	269 (13%)	478 (13%)
2016	116 (11%)	35 (5%)	311 (16%)	462 (13%)
2017 (up to June)	66 (6%)	12 (2%)	176 (9%)	254 (7%)
**p-value from chi-squared test**		<0.001	<0.001	
**Organism group**^c^				
*E*. *coli*	172 (17%)	113 (17%)	395 (20%)	680 (18%)
*Enterobacter*	94 (9%)	46 (7%)	167 (8%)	307 (8%)
*Klebsiella*	113 (11%)	58 (9%)	185 (9%)	356 (10%)
Other gram negative bacteria	89 (9%)	49 (7%)	182 (9%)	320 (9%)
Group B *Streptococcus*	79 (8%)	68 (10%)	154 (8%)	301 (8%)
*S*. *aureus*	243 (23%)	205 (30%)	451 (23%)	899 (24%)
*Enterococcus*	176 (17%)	87 (13%)	327 (17%)	590 (16%)
Other gram positive bacteria	10 (1%)	10 (1%)	31 (2%)	51 (1%)
Fungi	61 (6%)	44 (6%)	80 (4%)	185 (5%)
**p-value from chi-squared test**		0.006	0.128	

^a^ BSI caused by clearly pathogenic organisms with a sample date between two days after admission and two days following discharge

^b^ Babies with missing data for age/gestation excluded from percentages

^c^ For babies with more than one BSI, organism is from the first BSI

### BSI trends

Across all years, the addition of probabilistic linkage identified additional links (Table 2). Deterministic linkage captured 94% (750/796) of all babies that linked (deterministic and probabilistic linkage combined) in 2016–17 compared with 82% (589/722) in 2010–11. This reflects the increased recording of identifiers in later years (Fig 1).

**Table 2:** Number and proportion of babies with BSI caused by clearly pathogenic organisms between 2010–2017 and rate ratios (representing monthly rate change) based on Poisson regression for BSI identified through deterministic + probabilistic linkage, deterministic linkage alone, clinical records of BSI in NNRD and any record of BSI (either from linkage or clinical record of BSI in NNRD).

	Number of babies with BSI ^A^ (%)			Monthly rate ratio (95% CI)	Annual rate change
	Whole period Jan 10-Jun 17	Early period Jul 10- Jun 11	Late period Jul 16- Jun 17		
Deterministic and probabilistic links	5,629 (1.6%)	722 (1.7%)	796 (1.5%)	0.994 (0.987, 1.000)^B^	-7.5% (-14.3%, -0.1%)
Deterministic links	5,011 (1.4%)	589 (1.4%)	750 (1.4%)	0.996 (0.989, 1.003)	-4.9% (-12.4%, +3.1%)
Clinical record of BSI ^A^	3,277 (0.9%)	285 (0.7%)	450 (0.8%)	0.998 (0.990, 1.007)	-1.8% (-10.9%, +8.2%)
Any record of BSI ^A^	7,081 (2.0%)	860 (2.0%)	1,000 (1.8%)	0.994 (0.998, 1.000)^C^	-6.7% (-12.8%, -0.2%)
Total babies in NNRD	349,740	42,205	54,279		

^A^ BSI = BSI caused by a clearly pathogenic organism with sample date between 7 days before admission and 14 days after discharge; based on admissions to 112 NICUs and LNUs;

^B^ Upper limit of rate ratio is 0.9999188 therefore considered significant;

^C^ Upper limit of rate ratio is 0.999865 therefore considered significant

**Fig 3:**
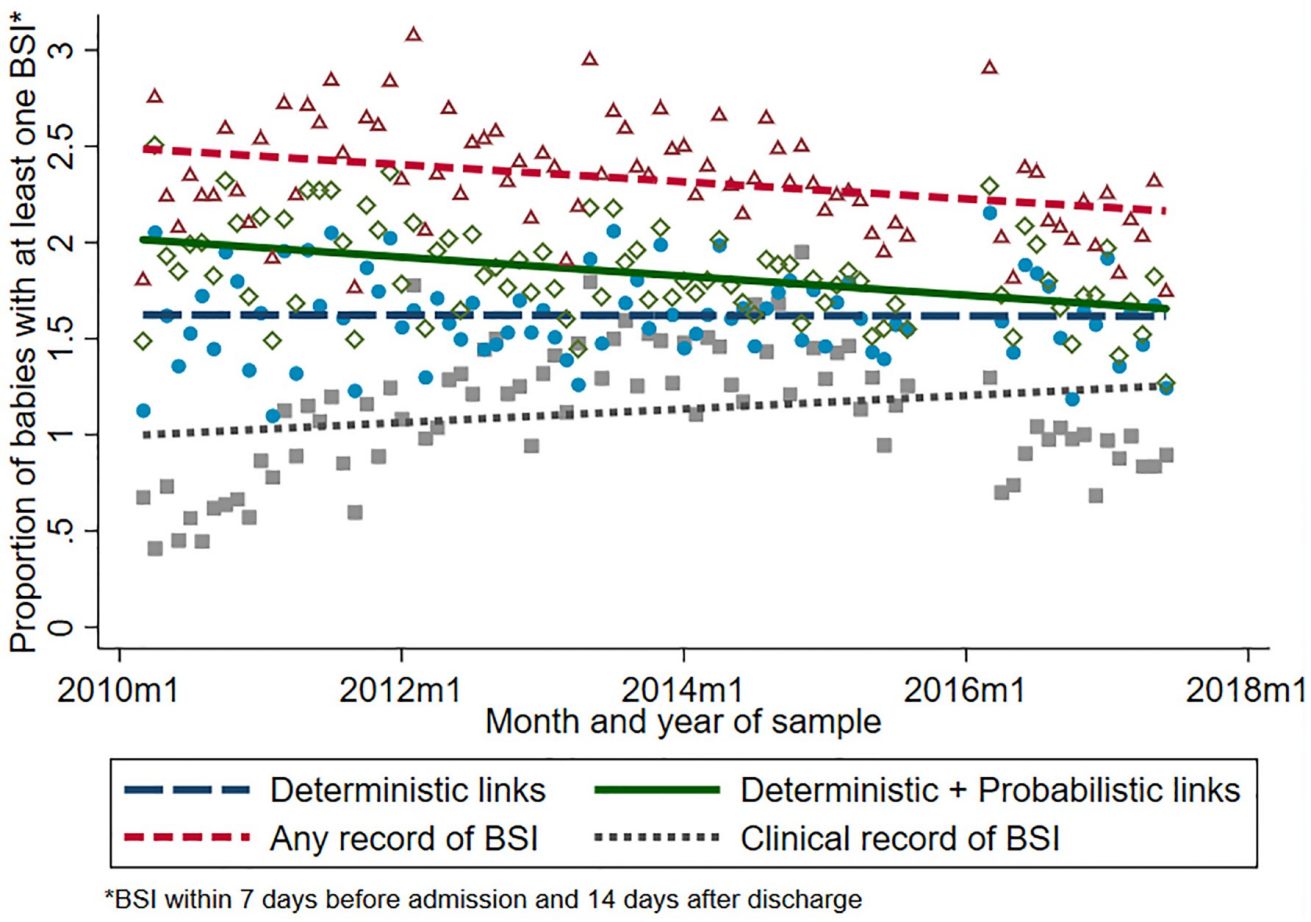
Monthly trend in the proportion of babies admitted to 112 NNUs from 2010–2017 with at least one BSI. Trends for i) BSI identified in deterministic and probabilistic linkage of NNRD and surveillance data (primary outcome measure), ii) BSI identified in deterministic linkage of NNRD and surveillance data, iii) any record of BSI (linkage or clinical records of BSI in NNRD), and iv) clinical record of BSI in NNRD alone. The lines are smoothed predicted estimates from Poisson models. BSI defined as pathogens isolated in blood or CSF samples between 7 days before and 14 days after NNU admission.

We found a decline in the proportion of babies in NNU who linked to a BSI over time with an annual decrease of -7.5% (95% CI: -14.3%, -0.1%) for the primary outcome, using deterministic and probabilistic linkage (Fig 3, Table 2). Whereas, deterministic linkage alone found a stable trend (annual decrease: -4.9%, 95% CI: -12.4%, +3.1%). The rate of any record of BSI also decreased over time, but more gradually than using the primary measure (annual decrease: -6.7%, 95% CI: -12.8, -0.2). The proportion of babies with BSI identified using only clinical records of BSI in NNRD did not significantly change over time and was always lower than the BSI rate using linkage with surveillance data (Table 2, Fig 3). The average number of BSI episodes per baby with BSI was 1.01 (6922 BSI in 6821 babies) for all babies and 1.02 (3524 BSI in 3441 babies) for babies <32 weeks gestation. In total, 97 (0.03%) babies experienced two BSI episodes, 81 of which were <32 weeks gestation, episodes and four babies experiencing three BSI episodes, 1 of which was <32 weeks gestation.

## Discussion

Linking electronic health records from 112 NNUs in England to BSI records from laboratory surveillance identified 5.0% of babies born before 32 weeks gestation who had at least one BSI caused by a clearly pathogenic organism during their NNU admission, compared to 2.9% using clinical records of BSI in NNRD alone. Our estimate increased to 5.9% when combining babies who linked to a BSI record from the surveillance data with unlinked clinical records of BSI in NNRD. The equivalent proportions for babies of all gestations were 1.0%, 0.6% and 1.2%, respectively. Linking both data sources that contain information on BSI improves detection of BSI compared with using either source alone, but may still underestimate BSI rates for early years when clinical records of BSI and patient identifiers in infection surveillance data were poorly completed. Deterministic linkage performed better in later years when patient identifiers were more complete. Despite improved data quality in the most recent years, probabilistic linkage continued to identify links that would have been missed using deterministic methods alone. The number of babies who linked to a BSI decreased by 7.5% per year using deterministic and probabilistic linkage. The trend in rate of BSI is more credible for deterministic plus probabilistic linkage than with deterministic linkage alone as the recording of NHS number improved over time.

A strength of this study is that we have data for 89% of LNUs and 96% of NICUs in England. This is the first study to show national trends in rates of BSI in NNUs across England. We have demonstrated the feasibility and challenges of linking the electronic patient health records to national infection surveillance system. Our methods, including careful attention to data quality, could be applicable to other countries as it is common for clinical data entered to electronic patient health records to be separate from data on BSI captured by laboratories.[18, 19]

A limitation of this study is that there were 1,452 (44% of 3,277) babies with a clinical record of BSI in NNRD for which we could not find a linked BSI record in the surveillance data. When restricted to BSI during NNU admission (two days after admission to two days after discharge), there were only 607 (35% of 1,734) babies with a clinical records of BSI for which we could not find a linked BSI record in the surveillance data, suggesting that babies with a clinical record of BSI which occurred within the first two days of admission (commonly first two days of life) were less likely to link than those with a BSI which occurred later. Three possible mechanisms could account for clinical records of BSI not linking to surveillance data. First, we may have missed links due to missing identifiers in the surveillance data. Second, true cases of BSI may have been reported to NNRD but not to the national laboratory infection surveillance system. Third, the clinical records of BSI in NNRD could be false positives due to data entry errors. It is likely that all three mechanisms were in effect, but we expect there were few false positives in the NNRD and therefore that most missed links were due to missing identifiers or unreported BSI in the national surveillance data. We believe the clinical records of BSI in NNRD are likely true as records include dates and organism cultured.

BSI records from the surveillance data that did not link to NNRD may be explained by BSI that did not occur during an admission to NNU. For example, some of the BSI may belong to the 3,000 babies aged <1 month admitted to a paediatric intensive care unit each year.[20] BSI records in surveillance data may also represent babies in the postnatal maternity ward, children’s ward or emergency departments. Linkage to hospital episode statistics (HES) would provide further information on the location of young babies with BSI who are not in NNU.[21] In this linkage study, it was difficult to evaluate linkage quality, since we did not expect all records from either dataset to link and therefore simple linkage (or match) rates are unhelpful. Our comparison of the characteristics of linked and unlinked records restricted to those we did expect to link (babies with a clinical record of BSI in NNRD) indicated that there were differences in ascertainment depending on age, year of sample and gestational age at birth. Certain pathogens were also more likely to link. This linkage, whilst imperfect, enhances the data in NNRD and provides improved ascertainment whilst placing no extra burden of data collection on staff in NNUs.

Our rates of BSI are lower than those reported by the National Neonatal Audit Programme (NNAP). We found a lower proportion of babies <32 weeks of gestation with BSI during their stay in NNU in our study (5%) compared with the 9% reported by the NNAP.[12] A major difference is that the NNAP included all BSI during NNU admission, whereas we excluded the first two days as they are likely early onset BSI or from a babies previous admission (e.g. postnatal ward). Although we both restricted our analyses to clearly pathogenic organisms, the exact organisms excluded differs as not all BSI in our surveillance data were reported in the NNAP report.

In the last decade, many initiatives have been implemented to reduce health-care acquired infections and therefore a decline in rates of BSI was expected and has been reported in UK NNUs.[17, 22–25] The NeonIN surveillance network reported a decline in BSI rate from 2005 to 2014 with the BSI rate (excluding coagulase negative *Staphylococcus*) per neonatal admission in 2014 around 2%.[17] This is half of our BSI rate during NNU days of stay, which is likely due to our inclusion of 112 NNUs across England (compared with 30 NNUs in NeonIn) and our restriction to clearly pathogenic organisms, which was more stringent than excluding coagulase negative *Staphylococcus*. NeonIN report a decline in the proportion of babies in NNU with any record of BSI, whereas we did not identify a significant trend. As missed links are more likely in earlier years due to higher levels of missing data, our ascertainment of BSI is likely to have improved over time, and the true trend may be a decline. The trends we report are not adjusted for risk factors or time at risk and therefore caution should be used when drawing clinical conclusions from the data. Similarly, the stable trend in the proportion of babies with BSI based on clinical records of BSI in NNRD alone can be explained by improved recording over time of data on positive blood and CSF cultures in NNUs. In 2016, only 25 of 181 (14%) NNUs in England, Wales and Scotland provided assurance to NNAP that they had entered complete data on all BSI but this increased to 74 of 179 (41%) in 2017.[12]

We have brought together clinical information from NNUs and national surveillance data on BSI to create a resource that can be used, and potentially updated, to evaluate risk-adjusted rates of BSI in NNUs in England. Our work highlights the importance of probabilistic linkage, as even in later years when data quality has improved, probabilistic linkage identified links not found using deterministic linkage alone. If completeness and accuracy of BSI data recorded in NNRD continues to improve, linkage to external datasets may not be required for monitoring trends. However our comparison of data sources suggests that enhancing the clinical recording of BSI in NNRD with linkage to national infection surveillance data doubles rate of BSI compared with using clinical records of BSI from NNRD alone.

## Supporting information

S1 Appendix List of organisms categorised as clearly pathogenic organisms. (DOCX)

S2 Appendix Rules for identifier cleaning. (DOCX)

S3 Appendix Linkage methods. (DOCX)

S1 Fig Flow diagram of linkage steps. (PDF)

S2 Fig The percentage of BSI that could not be linked due to missing identifiers by month. (PNG)

S1 Dataset Minimal data. The number of BSI per month from linkage and clinical records of BSI.
(XLS)

## References

[1] Rosenthal VD, Bijie H, Maki DG, Mehta Y, Apisarnthanarak A, Medeiros EA, et al International Nosocomial Infection Control Consortium (INICC) report, data summary of 36 countries, for 2004–2009. American journal of infection control. 2012;40(5):396–407. 10.1016/j.ajic.2011.05.020 . 21908073

[2] Wilson J, Elgohari S, Livermore DM, Cookson B, Johnson A, Lamagni T, et al Trends among pathogens reported as causing bacteraemia in England, 2004–2008. Clinical microbiology and infection: the official publication of the European Society of Clinical Microbiology and Infectious Diseases. 2011;17(3):451–8. 10.1111/j.1469-0691.2010.03262.x . 20491834

[3] Fraser C, Harron K, Dalton L, Gilbert R, Oddie SJ. Variation in infection prevention practices for peripherally inserted central venous catheters: A survey of neonatal units in England and Wales. PloS one. 2018;13(11):e0204894 Epub 2018/11/02. 10.1371/journal.pone.0204894 . 30383769 PMC6211675

[4] Ista E, van der Hoven B, Kornelisse RF, van der Starre C, Vos MC, Boersma E, et al Effectiveness of insertion and maintenance bundles to prevent central-line-associated bloodstream infections in critically ill patients of all ages: a systematic review and meta-analysis. The Lancet Infectious diseases. 2016;16(6):724–34. Epub 2016/02/26. 10.1016/S1473-3099(15)00409-0 . 26907734

[5] Helder O, van den Hoogen A, de Boer C, van Goudoever J, Verboon-Maciolek M, Kornelisse R. Effectiveness of non-pharmacological interventions for the prevention of bloodstream infections in infants admitted to a neonatal intensive care unit: A systematic review. Int J Nurs Stud. 2013;50(6):819–31. Epub 2012/03/06. 10.1016/j.ijnurstu.2012.02.009 . 22385913

[6] Payne V, Hall M, Prieto J, Johnson M. Care bundles to reduce central line-associated bloodstream infections in the neonatal unit: a systematic review and meta-analysis. Arch Dis Child Fetal Neonatal Ed. 2017 Epub 2017/11/28. 10.1136/archdischild-2017-313362 . 29175985

[7] Holmes A, Dore CJ, Saraswatula A, Bamford KB, Richards MS, Coello R, et al Risk factors and recommendations for rate stratification for surveillance of neonatal healthcare-associated bloodstream infection. The Journal of hospital infection. 2008;68(1):66–72. 10.1016/j.jhin.2007.08.019 . 17942191

[8] Couto RC, Pedrosa TM, Tofani Cde P, Pedroso ER. Risk factors for nosocomial infection in a neonatal intensive care unit. Infection control and hospital epidemiology. 2006;27(6):571–5. 10.1086/504931 . 16755475

[9] Gale C, Morris I, Neonatal Data Analysis Unit Steering B. The UK National Neonatal Research Database: using neonatal data for research, quality improvement and more. Arch Dis Child Educ Pract Ed. 2016;101(4):216–8. Epub 2016/03/13. 10.1136/archdischild-2015-309928 . 26968617 PMC4975807

[10] NHS England. Service Specifications: Neonatal Critical Care (Intensive Care, HDU and Special Care) 2015.

[11] Ismail AQT, Palmer K. Assessing the accuracy of the National Neonatal Audit Programme calculated central line-associated bloodstream infection rate from local data. Archives of disease in childhood Fetal and neonatal edition. 2017 10.1136/archdischild-2017-313291 . 28676562

[12] The National Neonatal Audit Programme. National Neonatal Audit Programme 2018 report on 2017 data. 2018.

[13] PHE. Laboratory reporting to Public Health England: A guide for diagnostic laboratories. 2016.

[14] Sayers A, Ben-Shlomo Y, Blom AW, Steele F. Probabilistic record linkage. International journal of epidemiology. 2015 10.1093/ije/dyv322 . 26686842 PMC5005943

[15] Harron K, Dibben C, Boyd J, Hjern A, Azimaee M, Barreto ML, et al Challenges in administrative data linkage for research. Big Data Soc. 2017;4(2):2053951717745678 Epub 2018/11/02. 10.1177/2053951717745678 . 30381794 PMC6187070

[16] Fellegi IP, Sunter AB. A Theory for Record Linkage. Journal of the American Statistical Association. 1969;64(328):1183–210. 10.1080/01621459.1969.10501049

[17] Cailes B, Kortsalioudaki C, Buttery J, Pattnayak S, Greenough A, Matthes J, et al Epidemiology of UK neonatal infections: the neonIN infection surveillance network. Archives of disease in childhood Fetal and neonatal edition. 2018;103(6):F547–F53. Epub 2017/12/07. 10.1136/archdischild-2017-313203 . 29208666

[18] Lim FJ, Blyth CC, Fathima P, de Klerk N, Moore HC. Record linkage study of the pathogen-specific burden of respiratory viruses in children. Influenza and other respiratory viruses. 2017;11(6):502–10. Epub 2017/10/11. 10.1111/irv.12508 . 28991397 PMC5705691

[19] Moore HC, de Klerk N, Keil AD, Smith DW, Blyth CC, Richmond P, et al Use of data linkage to investigate the aetiology of acute lower respiratory infection hospitalisations in children. Journal of paediatrics and child health. 2012;48(6):520–8. Epub 2011/11/15. 10.1111/j.1440-1754.2011.02229.x . 22077532 PMC7166791

[20] Harron K, Mok Q, Dwan K, Ridyard CH, Moitt T, Millar M, et al CATheter Infections in CHildren (CATCH): a randomised controlled trial and economic evaluation comparing impregnated and standard central venous catheters in children. Health technology assessment. 2016;20(18):vii–xxviii, 1–219. 10.3310/hta20180 . 26935961 PMC4809464

[21] NHS Digital. Hospital Episode Statistics (HES) 2019 [cited 2019 02/09/2019]. https://digital.nhs.uk/data-and-information/data-tools-and-services/data-services/hospital-episode-statistics#top.

[22] Bion J, Richardson A, Hibbert P, Beer J, Abrusci T, McCutcheon M, et al 'Matching Michigan': a 2-year stepped interventional programme to minimise central venous catheter-blood stream infections in intensive care units in England. BMJ Qual Saf. 2013;22(2):110–23. Epub 2012/09/22. 10.1136/bmjqs-2012-001325 . 22996571 PMC3585494

[23] Montgomery-Taylor S, Emery F, Anthony M. Eighteen months of "matching Michigan" at a UK neonatal intensive care unit. The Pediatric infectious disease journal. 2013;32(5):565–7. Epub 2013/01/24. 10.1097/INF.0b013e3182868389 . 23340554

[24] NHS. Saving Lives: reducing infection, delivering clean and safe care. 2007.

[25] Sinha AK, Murthy V, Nath P, Morris JK, Millar M. Prevention of Late Onset Sepsis and Central-line Associated Blood Stream Infection in Preterm Infants. The Pediatric infectious disease journal. 2016;35(4):401–6. Epub 2015/12/03. 10.1097/INF.0000000000001019 . 26629870

