# Supplementary material for: Linking surveillance and clinical data for evaluating trends in bloodstream infection rates in neonatal units in England
Source: PLoS One. 2019 Dec 12;14(12):e0226040. doi: 10.1371/journal.pone.0226040 (PMC6907823; doi:10.1371/journal.pone.0226040)
Supplement: S1 Appendix — (DOCX) [file pone.0226040.s001.docx]

**Table A: List of organisms classified as clearly pathogenic (included)**

| **Clearly pathogenic organisms** | | |
| --- | --- | --- |
| ANAEROCOCCUS PREVOTII | CANDIDA HAEMULONIS | PASTEURELLA HAEMOLYTICA |
| BACTEROIDES CAPILLOSUS | CANDIDA KRUSEI | PASTEURELLA MULTOCIDA |
| BACTEROIDES DISTASONIS | COCCIDIOIDES SP | PASTEURELLA OTHER NAMED |
| BACTEROIDES OVATUS | CRYPTOCOCCUS ALBIDUS | PASTEURELLA PNEUMOTROPICA |
| BACTEROIDES SP | CRYPTOCOCCUS SP | PROTEUS PENNERI |
| BACTEROIDES UNIFORMIS | MALASSEZIA FURFUR | PROTEUS SP |
| BACTEROIDES VULGATUS | MALASSEZIA PACHYDERMATIS | PROTEUS VULGARIS |
| CLOSTRIDIUM BEIJERINCKII | MALASSEZIA SP | PROVIDENCIA STUARTII |
| CLOSTRIDIUM BIFERMENTANS | RHODOTORULA OTHER NAMED | RAOULTELLA PLANTICOLA (KLEBSIELLA PLANTICOLA) |
| CLOSTRIDIUM BUTYRICUM | RHODOTORULA RUBRA | RAOULTELLA SP |
| CLOSTRIDIUM PARAPUTRIFICUM | RHODOTORULA SP | RAOULTELLA TERRIGENA |
| CLOSTRIDIUM SEPTICUM | AEROMONAS CAVIAE | SALMONELLA ABA |
| CLOSTRIDIUM SORDELLI | AEROMONAS HYDROPHILA | SALMONELLA AGAMA |
| CLOSTRIDIUM SP | AEROMONAS SALMONICIDA | SALMONELLA AJIOBO |
| CLOSTRIDIUM SPOROGENES | AEROMONAS SOBRIA | SALMONELLA APAPA |
| CLOSTRIDIUM TERTIUM | AEROMONAS SP | SALMONELLA ARIZONAE |
| FUSOBACTERIUM NECROPHORUM | CAMPYLOBACTER FETUS | SALMONELLA BRANDENBURG |
| FUSOBACTERIUM NUCLEATUM | CAMPYLOBACTER JEJUNI | SALMONELLA COLINDALE |
| FUSOBACTERIUM SP | CAMPYLOBACTER SP | SALMONELLA COTHAM |
| GARDNERELLA OTHER NAMED | CAMPYLOBACTER UREOLYTICUS | SALMONELLA CUBANA |
| GARDNERELLA VAGINALIS | CEDECEA LAPAGEI | SALMONELLA DJUGU |
| PEPTOSTREPTOCOCCUS ASACCHAROLYTICUS | CITROBACTER AMALONATICUS | SALMONELLA DUBLIN |
| PEPTOSTREPTOCOCCUS MAGNUS | CITROBACTER BRAAKII | SALMONELLA ENTERITIDIS |
| PEPTOSTREPTOCOCCUS OTHER NAMED | CITROBACTER FARMERI | SALMONELLA GOLD-COAST |
| PREVOTELLA BIVIA | ENTEROBACTER AGGLOMERANS (PANTOEA AGGLOMERANS) | SALMONELLA HADAR |
| PREVOTELLA BUCCALIS | ENTEROBACTER HORMAECHEI | SALMONELLA HEIDELBERG |
| PREVOTELLA ORALIS | ENTEROBACTER INTERMEDIUS (ENTEROBACTER INTERMEDIUM) | SALMONELLA HOFIT |
| STREPTOCOCCUS ANAEROBIC | ENTEROBACTER SAKAZAKII | SALMONELLA HULL |
| VEILLONELLA ATYPICA | ESCHERICHIA HERMANNII | SALMONELLA INFANTIS |
| VEILLONELLA NAMED | ESCHERICHIA OTHER NAMED | SALMONELLA KEDOUGOU |
| ASPERGILLUS FUMIGATUS | ESCHERICHIA SP | SALMONELLA KIAMBU |
| ASPERGILLUS NIGER | ESCHERICHIA VULNERIS | SALMONELLA KIBUSI |
| ASPERGILLUS OTHER NAMED | HAFNIA ALVEI | SALMONELLA KINTAMBO |
| ASPERGILLUS SP | KLEBSIELLA ORNITHNOLYTICA | SALMONELLA KISARAWE |
| CANDIDA CIFERRII | KLEBSIELLA OTHER NAMED | SALMONELLA MATOPENI |
| CANDIDA FAMATA | KLEBSIELLA PNEUMONIAE SUBSP OZENAE | SALMONELLA MISSISSIPPI |
| SALMONELLA MONSCHAUI | SALMONELLA NEWPORT | SALMONELLA MUENSTER |
| SALMONELLA MONTEVIDEO | SALMONELLA ORANIENBURG | PANTOEA SEPTICA |
| SALMONELLA MUENCHEN | SALMONELLA POONA | PANTOEA SP |
| SALMONELLA READING | ENTEROCOCCUS GALLINARUM | PSEUDOMONAS AERUGINOSA |
| SALMONELLA SAPHRA | ENTEROCOCCUS HIRAE | HAEMOPHILUS INFLUENZAE |
| SALMONELLA SENFTENBERG | ENTEROCOCCUS SP | PASTEURELLA SP |
| SALMONELLA SINSTORF | STAPHYLOCOCCUS AUREUS | NEISSERIA MENINGITIDIS |
| SALMONELLA SP | LISTERIA MONOCYTOGENES | BACTEROIDES FRAGILIS |
| SALMONELLA STANLEY | CITROBACTER DIVERSUS (C. KOSERI) | CLOSTRIDIUM PERFRINGENS |
| SALMONELLA TEL-EL-KEBIR | CITROBACTER FREUNDII | CANDIDA ALBICANS (STELLATOIDEA) |
| SALMONELLA TYPHI AND PARATYPHI | CITROBACTER OTHER NAMED | CANDIDA GLABRATA |
| SALMONELLA TYPHIMURIUM | CITROBACTER SP | CANDIDA OTHER NAMED |
| SALMONELLA UNNAMED | COLIFORM | CANDIDA TROPICALIS |
| SALMONELLA VIRCHOW | ENTEROBACTER AEROGENES | CANDIDA DUBLINIENSIS |
| SALMONELLA VITKIN | ENTEROBACTER AGGLOMERANS | CANDIDA GUILLIERMONDII |
| SALMONELLA WICHITA | ENTEROBACTER AMNIGENUS | CANDIDA PARAPSILOSIS |
| SERRATIA PLYMUTHICA | ENTEROBACTER ASBURIAE | CANDIDA FABIANII |
| SERRATIA PROTEAMACULAS | ENTEROBACTER CLOACAE | CANDIDA LUSITANIAE |
| SERRATIA RUBIDAEA | ENTEROBACTER CLOACAE COMPLEX | CANDIDA SP |
| SHIGELLA FLEXNERI | HANSENULA SP | ENTEROCOCCUS DURANS |
| YERSINIA ENTEROCOLITICA | ENTEROBACTER GERGOVIAE | ENTEROCOCCUS FAECALIS |
|  | ENTEROBACTER KOBEI | ENTEROCOCCUS FAECIUM |
| ENTEROCOCCUS AVIUM | ENTEROBACTER OTHER NAMED | PASTEURELLA SP |
| ENTEROCOCCUS CASSELIFLAVUS | ENTEROBACTER SP | PROTEUS MIRABILIS |
| ENTEROCOCCUS FAECALIS (STREPTOCOCCUS FAECALIS) | ESCHERICHIA COLI | PROVIDENCIA ALCALIFACIENS |
| ENTEROCOCCUS RAFFINOSUS | KLEBSIELLA AEROGENES | RAOULTELLA PLANTICOLA |
| LISTERIA SP | KLEBSIELLA ORNITHNOLYTICA | SERRATIA LIQUEFACIENS |
| STREPTOCOCCUS GROUP A STEM | KLEBSIELLA OXYTOCA | SERRATIA MARCESCENS |
| STREPTOCOCCUS GROUP B STEM | KLEBSIELLA PNEUMONIAE | SERRATIA ODORIFERA |
| STREPTOCOCCUS GROUP C STEM | KLEBSIELLA SP | SERRATIA OTHER NAMED |
| STREPTOCOCCUS GROUP D STEM | KLUYVERA SP | SERRATIA SP |
| STREPTOCOCCUS GROUP G STEM | LECLERCIA ADECARBOXYLATA | ACINETOBACTER BAUMANNII |
| STREPTOCOCCUS MILLERI GROUP | MORGANELLA MORGANII | STREPTOCOCCUS INTERMEDIUS GROUP |
| STREPTOCOCCUS PNEUMONIAE |  |  |

**Table B: List of other organisms (excluded)**

| **Other organisms** | | |
| --- | --- | --- |
| STAPHYLOCOCCUS COAGULASE NEGATIVE | CORYNEBACTERIUM JEIKEIUM (JK) | GORDONIA SP |
| STREPTOCOCCUS ALPHA AND NON-HAEMOLYTIC | BURKHOLDERIA GLADIOLI | MICROCOCCUS LYLAE |
| PSEUDOMONAS SP | NEISSERIA POLYSACCHAREAE | STEPHANOASCUS CIFERRII |
| STAPHYLOCOCCUS OTHER NAMED | RAHNELLA SP | STOMATOCOCCUS SP |
| PROPIONIBACTERIUM FREUDENREICHII | HAEMOPHILUS APHROPHILUS | HAEMATOBACTER SP |
| MICROCOCCUS SP | ACHROMOBACTER XYLOSOXIDANS | STREPTOCOCCUS PERORIS |
| STREPTOCOCCUS SP | EGGERTHELLA LENTA (EUBACTERIUM LENTUM) | OCHROBACTRUM SP |
| BACILLUS CEREUS | MICROCOCCUS OTHER NAMED | MICROBACTERIUM SP |
| ACINETOBACTER LWOFFII | BREVIBACTERIUM OTHER NAMED | GORDONIA BRONCHIALIS (RHODOCOCCUS BRONCHIALIS) |
| MORAXELLA CATARRHALIS | ACTINOMYCES ODONTOLYTICUS | KOCURIA ROSEA |
| CORYNEBACTERIUM SP | BACILLUS PUMILUS | RUMINOCOCCUS GNAVUS |
| DIPHTHEROIDS | ROSEOMONAS GILARDII | AEROCOCCUS URINAE |
| STREPTOCOCCUS OTHER NAMED | NEISSERIA FLAVESCENS | PARACOCCUS YEEII |
| BACILLUS SP | ROTHIA DENTOCARIOSIA | CORYNEBACTERIUM PROPINQUUM |
| OCHROBACTRUM ANTHROPI | CHRYSEOBACTERIUM MENINGOSEPTICUM | CORYNEBACTERIUM COYLEAE |
| STREPTOCOCCUS INTERMEDIUS GROUP | KOCURIA KRISTINAE | LACTOBACILLUS PARACASEI |
| ACINETOBACTER SP | ABIOTROPHIA DEFECTIVA | CORYNEBACTERIUM SIMULANS |
| STAPHYLOCOCCUS SP | PSEUDOMONAS ORYZIHABITANS (FLAVIMONAS ORYZIHABITANS) | CORYNEBACTERIUM AURIS |
| LEUCONOSTOC SP | RHODOCOCCUS SP | ACINETOBACTER URSINGII |
| STENOTROPHOMONAS MALTOPHILIA | KOCURIA SP | BACILLUS SILVESTRIS |
| AEROCOCCUS VIRIDANS | CORYNEBACTERIUM OTHER NAMED | STAPHYLOCOCCUS VITULINUS |
| AEROCOCCUS OTHER NAMED | ACTINOMYCES NAESLUNDII | HAEMOPHILUS HAEMOLYTICUS |
| AEROCOCCUS SP | RHIZOBIUM RADIOBACTER (AGROBACTERIUM TUMEFACIENS) | PSEUDOMONAS OLEOVORANS |
| MORAXELLA SP | NEISSERIA LACTAMICA | STREPTOCOCCUS INFANTIS |
| CORYNEBACTERIUM STRIATUM | LACTOBACILLUS LACTIS | ACINETOBACTER RADIORESISTENS |
| NEISSERIA SP | RALSTONIA PICKETTII | ROSEOMONAS MUCOSA |
| STREPTOCOCCUS VESTIBULARIS | SPHINGOMONAS SP | BREVIBACTERIUM CASEI |
| STREPTOCOCCUS OTHER GROUP (NOT A-D F G) | CORYNEBACTERIUM MINUTISSIMUM | LACTOBACILLUS GASSERI |
| PSEUDOMONAS PAUCIMOBILIS (SPHINGOMONAS PAUCIMOBILIS) | BREVUNDIMONAS DIMINUTA | BIFIDOBACTERIUM LONGUM |
| EIKENELLA CORRODENS | HAEMOPHILUS PARAHAEMOLYTICUS | OCEANOBACILLUS PROFUNDUS |
| HAEMOPHILUS SP | CAPNOCYTOPHAGA OTHER NAMED | ACTINOMYCES SP |
| PSEUDOMONAS OTHER NAMED | PHIALOPHORA OTHER NAMED | ACIDOVORAX TEMPERANS |
| LACTOCOCCUS LACTIS | CHRYSEOBACTERIUM SP | BIFIDOBACTERIUM SP |
| BURKHOLDERIA CEPACIA | STENOTROPHOMONAS SP | ROTHIA AERIA |
| ACHROMOBACTER SP | LACTOBACILLUS FERMENTUM | PSEUDOMONAS ALCALIGENES |
| AURANTIMONAS ALTAMIRENSIS | ACTINOMYCES CARDIFFENSIS | PARABACTEROIDES DISTASONIS |
| CORYNEBACTERIUM PSEUDODIPHTHERITICUM | GEOTRICHUM SP | BACILLUS SUBTILIS |
| HAEMOPHILUS PARAINFLUENZAE | MORAXELLA NONLIQUEFACIENS | MICROCOCCUS VARIANS (KOCURIA VARIANS) |
| MICROCOCCUS LUTEUS (SARCINA) | CORYNEBACTERIUM AMYCOLATUM | KINGELLA SP |
| BREVIBACTERIUM SP | BACILLUS LICHENIFORMIS | KINGELLA DENITRIFICANS |
| STREPTOCOCCUS SOBRINUS | LACTOBACILLUS OTHER NAMED | PAENIBACILLUS GLUCANOLYTICUS |
| NEISSERIA OTHER NAMED | RHODOCOCCUS OTHER NAMED | BACILLUS CIRCULANS |
| BREVUNDIMONAS VESICULARIS | COLLINSELLA AEROFACIENS | ACINETOBACTER PARVUS |
| KINGELLA KINGAE | STOMATOCOCCUS MUCILAGINOSUS | PAENIBACILLUS PABULI |
| STREPTOCOCCUS ALACTOLYTICUS | STREPTOCOCCUS INFANTARIUS SUBSP NOV | PAENIBACILLUS AMYLOLYTICUS |
| GEMELLA MORBILLORUM | DERMABACTER HOMINIS | CORYNEBACTERIUM DIPHTHERIAE |
| MICROSPORUM SP | STREPTOCOCCUS LUTETIENSIS | STREPTOCOCCUS CRISTATUS |
| MORAXELLA OSLOENSIS | PROPIONIBACTERIUM ACNES | MICROBACTERIUM AURUM |
| ALCALIGENES FAECALIS | MASSILIA TIMONAE | PSEUDOMONAS LUTEOLA |
| PSEUDOMONAS STUTZERI | ACTINOMYCES OTHER NAMED | LYSINIBACILLUS SP |
| NEISSERIA SICCA | LACTOCOCCUS GARVIEAE | LACTOBACILLUS SP |
| GLOBICATELLA SANGUIS | LACTOBACILLUS JENSENII | LACTOBACILLUS CRISPATUS |
| GRANULICATELLA ADIACENS (ABIOTROPHIA ADJACENS) | BIFIDOBACTERIUM NAMED | PARACOCCUS SP |
| ARTHROBACTER SP | METHYLOBACTERIUM SP | HAEMOPHILUS PARAPHROHAEMOLYTICUS |
| ROTHIA SP | ROSEOMONAS SP | PSEUDOCLAVIBACTER SP |
| STREPTOCOCCUS GORDONII | CORYNEBACTERIUM AFERMENTANS | BREVIBACILLUS PARABREVIS |
| ACINETOBACTER JUNII | CHRYSEOBACTERIUM INDOLOGENES | STREPTOCOCCUS THERMOPHILUS |
| BIFIDOBACTERIUM BREVE | ACINETOBACTER JOHNSONII | ACTINOMYCES ORIS |
| ACINETOBACTER CALCOACETICUS (ANITRATUS) | DELFTIA ACIDOVORANS (COMAMONAS ACIDOVORANS) | HANSENULA SP |
| BREVUNDIMONAS SP | COMAMONAS TESTOSTERONI | CORYNEBACTERIUM MUCIFACIENS |
| LACTOBACILLUS RHAMNOSUS | DERMACOCCUS SP | STREPTOCOCCUS PSEUDOPORCINUS |
| RAHNELLA NAMED | MORAXELLA LACUNATA | STENOTROPHOMONAS ACIDAMINIPHILA |
| PEPTOCOCCUS SP | STAPHYLOCOCCUS PETTENKOFERI | SPHINGOBACTERIUM MULTIVORUM |
| GEMELLA HAEMOLYSANS | ELIZABETHKINGIA SP | BIFIDOBACTERIUM CATENULATUM |
| PSEUDOMONAS PUTIDA | CORYNEBACTERIUM AURIMUCOSUM | PSEUDOXANTHOMONAS KAOHSIUNGENSIS |
| LACTOCOCCUS SP | GRANULICATELLA ELEGANS | NEISSERIA CINEREA |
| PSEUDOMONAS FLUORESCENS | ABIOTROPHIA OTHER NAMED | NEISSERIA PERFLAVA |
| ACINETOBACTER OTHER NAMED | KOCURIA RHIZOPHILA | ACINETOBACTER HAEMOLYTICUS |
| BACILLUS OTHER NAMED | CORYNEBACTERIUM IMITANS | ACTINOMYCES VISCOSUS |
| HAEMOPHILUS OTHER NAMED | PAENIBACILLUS SP | BIFIDOBACTERIUM ADOLESCENTIS |
| ARCANOBACTERIUM HAEMOLYTICUM | NEISSERIA SUBFLAVA | MICROBACTERIUM PARAOXYDANS |
| ALCALIGENES SP | ELIZABETHKINGIA MIRICOLA | ACTINOMYCES NEUII |
| NEISSERIA MUCOSA | PEDIOCOCCUS ACIDILACTICI | LACTOCOCCUS CREMORIS |

| **Organisms not plausible to have been isolated from blood** |
| --- |
| TREPONEMA PALLIDUM |
| MYCOBACTERIUM TUBERCULOSIS |
| TREPONEMA SP |
| BORDETELLA SP |
| BORDETELLA PERTUSSIS |
| MYCOBACTERIUM SP |
| CLOSTRIDIUM DIFFICILE |
| MYCOBACTERIUM CHELONAE |
| MYCOBACTERIUM OTHER NAMED |
| BORDETELLA PARAPERTUSSIS |
| MYCOBACTERIUM FLAVESCENS |
| NEISSERIA GONORRHOEAE |

**Table C: List of organisms classified as not plausible (excluded)**
