# Supplementary material for: Linking surveillance and clinical data for evaluating trends in bloodstream infection rates in neonatal units in England
Source: PLoS One. 2019 Dec 12;14(12):e0226040. doi: 10.1371/journal.pone.0226040 (PMC6907823; doi:10.1371/journal.pone.0226040)
Supplement: S2 Appendix — (DOCX) [file pone.0226040.s002.docx]

# Rules for identifier cleaning

## I) Cleaning of NNRD

### Identifying babies in NNU

The Badger ID is used to identify babies within NNR and the patient ID is used to identify admissions. A baby will have the same Badger ID for all admissions but a new patient ID every time they are transferred. Some hospitals record all births in the Badger net system and therefore all babies are present in NNRD, whether or not they were admitted to NNU. Only babies cared for in NNU are of interest, so any records without at least one day with ‘location of care’ as NNU are removed.

### De-duplication

In babies with multiple admissions and missing data for baseline characteristics (e.g. patient identifiers, gestation, birthweight), data was copied from admissions to other NNUs to fill in the missing data.

Each baby should have one unique Badger ID. We checked that there were not different babies with the same Badger ID or the same baby with multiple Badger IDs. We examined agreement on patient identifiers to check records with the same Badger ID belonged to the same baby. If two records with the same Badger ID had any of the following, one was given new Badger ID

1. Different NHS number and different date of birth
2. Different NHS number and different mother’s NHS number
3. Different NHS number, birthweight and time of birth but same mother’s NHS number, date of birth and gestation (these were recorded as twins)

Within some Badger IDs, there was disagreement on one identifier but we still believed them to be the same baby if everything else agreed. This applied to sex and postcode (prefix, suffix or both). This meant there would be two identifiers for some babies. To avoid duplicates, babies were given a second postcode prefix or suffix. If all identifiers other than sex were the same, the most common entry for sex was set for all records of that baby. If there were an equal number for each sex, sex was set as missing because it could not be determined which was correct.

If date of birth was in disagreement and one was clearly invalid, for example a year away from all other dates (admission, discharge, rupture of membranes), this date of birth would be removed and only one date of birth would remain and replace the invalid date of birth. In all other cases where there was two dates of birth, a second date of birth variable was generated.

Keeping both identifiers when it is not clear which is correct is important because we do not know which one would be recorded in the PHE infection surveillance data. In the cases of postcode, both may be correct as postcode can change if someone moves house or a baby could have two postcodes if the parents live separately.

**NHS number**

NHS number was checked using the Modulus 11 test, 11% (48,001/432,029) of admissions with complete NHS number were invalid.

### Date of birth

Date of birth was well completed. In cases of missing date of birth we imputed date of birth using the following rules. Where available, date of birth was set to equal admission date because 75% of babies in the data are admitted on their day of birth and 90% are admitted in the first two days of life. If admission date was missing, we imputed from the first day of care. If admission date and care dates were not available, but the date of rupture of membranes was available we set the date of birth and admission date to the date of rupture of membranes. 91% of babies with complete date of rupture of membrane and date of birth have a date of rupture of membranes within plus or minus two days of date of birth therefore we believe it is reasonable to use rupture of membrane as a proxy for date of birth. Where date of birth, admission date, care date and date of ruptured membranes was missing but anonymised birth month and year was present, we set date of birth to 15th of each month (16,731 babies changed). For babies that have a stay in an NNU that spans two or more months, the date of birth was approximated using the anonymised day (minutes from birth). We created an indicator so we are able to identify babies with a proxy or imputed day of birth.

### Postcode

Special characters and spaces were removed from postcode and all letters were set to capitals. Postcodes that contained only a prefix or suffix were retained. Postcodes that contained a valid postcode followed by extra numbers were trimmed so only the valid postcode remained. All other invalid postcodes were set to missing. Postcode was split into prefix and suffix.

Valid prefix formats are: A1, A11, AA1, AA11, A1A, AA1A. Where 1 represents a number 0-9 and A represents a letter A-Z. The only valid postcode suffix is 1AA. Some babies had multiple postcodes recorded, this could be correct e.g. if the parents lived separately.

## II) Cleaning of SGSS

### Infection episodes

Infections are grouped into organism patient infection episodes, where multiple positive results for the same organism from a patient within a set time period (14 days for all organisms present in our extract) are classified as one infection episode. Categorising infection episodes is part of the standard processing of data when it is entered into SGSS, however there are often errors. We checked that babies with multiple cultures of the same organism within 14 days from the first sample were classed as one episode, and that no episodes contained different patients, or different organisms, or samples more than 14 days apart. We excluded mixed samples. Mixed samples were defined as more than one organism cultured form the same sample type (blood or CSF) on the same day from the same patient.

### NHS number

NHS number was checked to ensure all were 10 digit numbers and tested using the Modulus 11 test.

### Date of birth

All babies had date of birth present as they were selected based on age <1 year, which is calculated from the date of birth.

### Postcode

Postcode was checked and split to prefix and suffix following the same rules as used in cleaning of the NNRD.
