# Supplementary material for: Linking surveillance and clinical data for evaluating trends in bloodstream infection rates in neonatal units in England
Source: PLoS One. 2019 Dec 12;14(12):e0226040. doi: 10.1371/journal.pone.0226040 (PMC6907823; doi:10.1371/journal.pone.0226040)
Supplement: S3 Appendix — (DOCX) [file pone.0226040.s003.docx]

# Linkage methods

## Rules of linkage

Deterministic and probabilistic linkage followed a set of rules, shown in Table A.

**Table A: Rules for deterministic (D) and probabilistic (P) linkage**

| **Characteristic*** | **Conditions** |
| --- | --- |
| One to many (D, P) | Each BSI can only link to one admission but each admission can have multiple episodes of BSI. Therefore BSI that linked on NHS number were excluded in probabilistic stage |
| Blocking (P) | To reduce the number of comparison pairs we used blocking, only comparing records if they agreed on at least one of postcode prefix, postcode suffix or date of birth. |
| Dates (D, P) | Sample date (SGSS) falls within 7 days before admission and 14 days after discharge dates (NNRD) |
| NHS number (D) | 10 digit unique identifier assigned to babies at birth, present in both datasets |
| Postcode prefix (P) | Derived from postcode in both datasets. Accepted formats of prefix are: A1, A11, AA1, AA11, A1A, AA1A |
| Postcode suffix (P) | Derived from postcode in both datasets. Accepted format is 1AA |
| Date of birth (P) | Coded as DD/MM/YYYY in both datasets. Estimate imputed from other dates in NNRD where missing (see cleaning file) |
| Sex (P) | Coded as 1=male 2=female in both datasets. |
| Hospital and lab (P) | Hospital was poorly recorded in SGSS but laboratory was complete and most hospitals send their samples to one (or two) laboratories. Therefore we assigned each hospital in the neonatal clinical data the laboratory that it most frequently reported to using the NHS number links. Where there were two laboratories that a hospital commonly reported to we allowed two laboratories to be classified as agreement for one hospital. Therefore agreement on hospital was agreement between hospital and laboratory. |

**Fig A: The date restriction for links, if a sample date fell within the green period (7 days before admission to 14 days after discharge) it would be included and would be excluded in the grey period**

## Weight calculation

Match weights were calculated as $\log_{2} (\frac{m-probability}{u-probability})$ (Table B). The m-probability was calculated as the probability that a pair agree, disagree or the identifier is missing in either or both of the datasets, given the BSI record belongs to the admission. The u-probability was calculated as the probability that a pair agree, disagree or the identifier is missing in either or both of the datasets, given records belong to different individuals.

Match weights were totalled across identifiers to produce an overall weight for each comparison pair, based on the pattern of agreement of identifiers. A plot of the log frequency of the weights was examined to determine an upper and lower threshold, above which all pairs are classified as links and below which all pairs are classified as non-links. The agreement patterns of the weights close to the thresholds were examined and a second wider set of thresholds was selected to reduce potential missed matches and false matches.

**Table B: M probabilities (m), u probabilities (u) and match weights (MW) for agreement, missing or disagreement on each identifier**

| Identifier | Agree | | | Disagree | | | Missing | | | |
| --- | --- | --- | --- | --- | --- | --- | --- | --- | --- | --- |
|  | **m** | **u** | **MW** | **m** | **u** | **MW** | **m** | **u** | **MW** |  |
| Prefix | 0.8314 | 0.0528 | 3.9769 | 0.0505 | 0.6786 | -3.7482 | 0.1181 | 0.2686 | -1.1855 |  |
| Suffix | 0.7922 | 0.0217 | 5.1901 | 0.0869 | 0.7088 | -3.0280 | 0.1210 | 0.2695 | -1.1553 |  |
| Date of birth | 0.9978 | 0.9283 | 0.1042 | 0.0022 | 0.0715 | -5.3119 | 0.0000 | 0.0002 | 0.0000 |  |
| Sex | 0.9957 | 0.4646 | 0.9635 | 0.0018 | 0.4907 | -4.1603 | 0.0025 | 0.0447 | -2.3841 |  |
| Laboratory/  Hospital | 0.9060 | 0.0399 | 4.5051 | 0.0940 | 0.9406 | -3.3228 | 0.0000 | 0.0195 | 0.0000 |  |

## Selecting thresholds

We examined the plot of frequency of summed weights (Fig B) and selected the lower threshold as 2, below which all were non-links, and the upper threshold as 7, above which all were links.


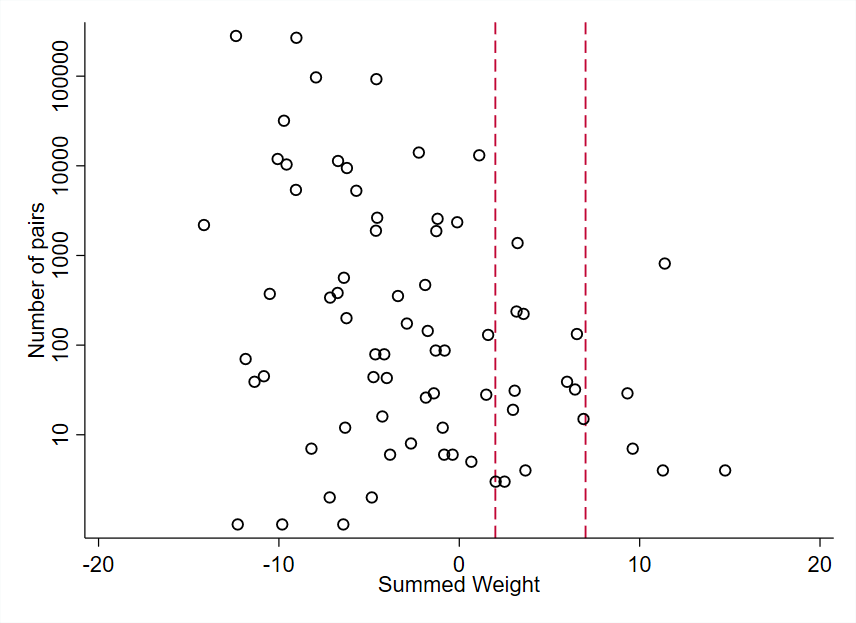


**Fig B: Frequency of summed weights showing lower and upper thresholds at 2 and 7, respectively**

## Multiple births

We used the same match weights and rules for manual review for multiple births as for singletons but manually reviewed the records separately as babies born on the same day to the same mother share most of their identifiers. In the few occasions where it was not possible to determine which baby a BSI should match to, we randomly allocated the BSI to one of the babies to avoid duplicates. Multiple births were identified in NNRD using the foetus number, birth order, date of birth and mother’s NHS number.

## Rules for manual review

The rules outlined in table Table C were applied to manual review to ensure consistency in decisions. To aid decision making, any clinical records indicating BSI and the daily record of suspected sepsis were checked in the NNRD as evidence that the baby had an infection. Where date of birth was in disagreement, the difference between the dates was examined. Where postcode prefix or suffix disagreed, the number of letters that matched was recorded e.g. RG1 and RK1 would be 2, RG1 and NG2 would be 1.

**Table C: The rules followed in manual review**

| Agreement on identifiers*  (1= agree, 0 = disagree, . = missing) | | | | | Condition | Allocate |
| --- | --- | --- | --- | --- | --- | --- |
| Prefix | Suffix | DOB** | Sex | Hospital |  |  |
| Any | | | | | BSI links to any baby with a match weight above the upper threshold | Non-link |
| 1 | 1 | 1 | 0 | . | BSI does not link to a different baby with a higher summed weight | Link |
| 1 | 0 | . | 1 | 1 | BSI does not link to a different baby with a higher summed weight and baby has a clinical record of BSI with the same organism group and/or specimen date | Link |
| 1 | 0 | . | 1 | 1 | BSI does not link to a different baby with a higher summed weight, DOB is recorded in one of the two datasets (missing in other) and age (in dataset with complete DOB) is <28 days old | Link |
| 1 | 0 | . | 1 | 1 | BSI does not link to a different baby with a higher summed weight and postcode suffix matches on at least one character | Link |
| 1 | 1 | 0 | 0 | 1 | BSI does not link to a different baby with a higher summed weight and age at BSI in surveillance data is <28 days old and difference between date of birth is <30 days | Link |
| 1 | 1 | 0 | 0 | 1 | BSI does not link to a different baby with a higher summed weight and baby has a clinical record of BSI with the same organism group and specimen date | Link |
| 0 | 1 | 1 | 0 | 1 | BSI does not link to a different baby with a higher summed weight and prefix shares at least two digits | Link |
| 1 | 1 | 1 | 0 | 0 | BSI does not link to a different baby with a higher summed weight and hospital in NNRD is geographically close to laboratory in surveillance data | Link |
| 1 | 0 | 1 | 0 | 1 | BSI does not link to a different baby with a higher summed weight and age at BSI in surveillance data is <28 days old | Link |
| 1 | 0 | 1 | 0 | 1 | BSI does not link to a different baby with a higher summed weight and postcode suffix matches on at least one character | Link |
| 1 | 0 | 1 | 0 | 1 | BSI does not link to a different baby with a higher summed weight and baby has a clinical record of BSI with the same organism group and/or specimen date | Link |
| 1 | 0 | 1 | 1 | 1 | BSI does not link to a different baby with a higher summed weight | Link |
| 1 | . | 0 | 1 | 1 | BSI does not link to a different baby with a higher summed weight and date difference is <30 days and age at BSI in surveillance data is <28 days old | Link |
| . | . | 1 | 1 | 1 | BSI does not link to a different baby with a higher summed weight and age at BSI in surveillance data is <28 days old | Link |
| . | . | 1 | 1 | 1 | BSI does not link to a different baby with a higher summed weight and baby has a clinical record of BSI with the same organism group and/or specimen date | Link |
| . | . | 1 | 1 | 1 | BSI does not link to a different baby with a higher summed weight and does not meet previous criteria (<28 days old/organism match/specimen date match) | Missing |
| . | . | 1 | . | … | Any given BSI does not link to another baby | Missing |
| . | . | 1 | . | 0 | Any given BSI does not link to another baby | Missing |
| . | . | 1 | 0 | . | Any given BSI does not link to another baby | Missing |
| . | . | 1 | 0 | 0 | Any given BSI does not link to another baby | Missing |
| . | . | 1 | 0 | 1 | Any given BSI does not link to another baby | Missing |
| 0 | . | 1 | . | 0 | Any given BSI does not link to another baby | Missing |
| 0 | . | 1 | 0 | . | Any given BSI does not link to another baby | Missing |
| 0 | . | 1 | 1 | . | Any given BSI does not link to another baby | Missing |
| . | . | 1 | 1 | . | Any given BSI does not link to another baby | Missing |
| . | . | 1 | 1 | 0 | Any given BSI does not link to another baby | Missing |

*The agreement on identifiers represents how much identifiers agreed for each comparison pair, where 1 indicates agreement on a given identifier, 0 indicates disagreement, and “.” indicates the identifier was missing in one or both datasets. **DOB = date of birth

## Extra linkage step for unlinked clinical records of BSI

Of 2,130 babies with a clinical record of BSI caused by a clearly pathogenic organism in NNRD before 28 days of age, 59% (1,261) linked to a BSI record from the surveillance data. I performed an additional linkage step on the remaining 869 babies with unlinked clinical records of BSI caused by a clearly pathogenic organism before 28 days of age. A BSI caused by a clearly pathogenic organism was reported to the corresponding laboratory in the month of the sample of the clinical record of BSI for 95% (822/869) of the babies (Table D). Only 45% (370/822) of the babies that matched a BSI record on sample month and laboratory-hospital matched a BSI with sample date within seven days before admission to NNU and 14 days after discharge. Only 176 babies were paired with a BSI record that had agreed on more than one of postcode prefix, postcode suffix, date of birth, sex, sample date or organism group. Through manual review, I identified that 57 babies (8% of 869 babies with unlinked clinical records of BSI) had an additional link to a BSI. If I was to assume that all 176 babies were true to links to those BSI records they agreed on at least one variable, the maximum linkage rate for babies with clinical records of BSI before 28 days of age would be 67% (1,261 + 176 / 2,130). This suggests that the issue is due to either missing data preventing linkage, or BSI that were recorded in NNRD but not reported to the infection surveillance data.

**Table D: The number of records* that linked in the additional linkage step**

|  | **Babies** |
| --- | --- |
| **Unlinked clinical records of BSI** | 869 |
| **Agreed with any surveillance record on month of sample and lab** | 822 |
| **Within date range** | 370 |
| **Agreed on at least two identifiers ^**^** | 176 |
| **Additional links identified in manual review** | 57 |

^*^unlinked clinical records of BSI from NNRD <28 days; ^**^Postcode prefix, postcode suffix, date of birth, sex, sample date or organism group
