# Supplementary figures and images for: Linking surveillance and clinical data for evaluating trends in bloodstream infection rates in neonatal units in England
Source: PLoS One. 2019 Dec 12;14(12):e0226040. doi: 10.1371/journal.pone.0226040 (PMC6907823; doi:10.1371/journal.pone.0226040)

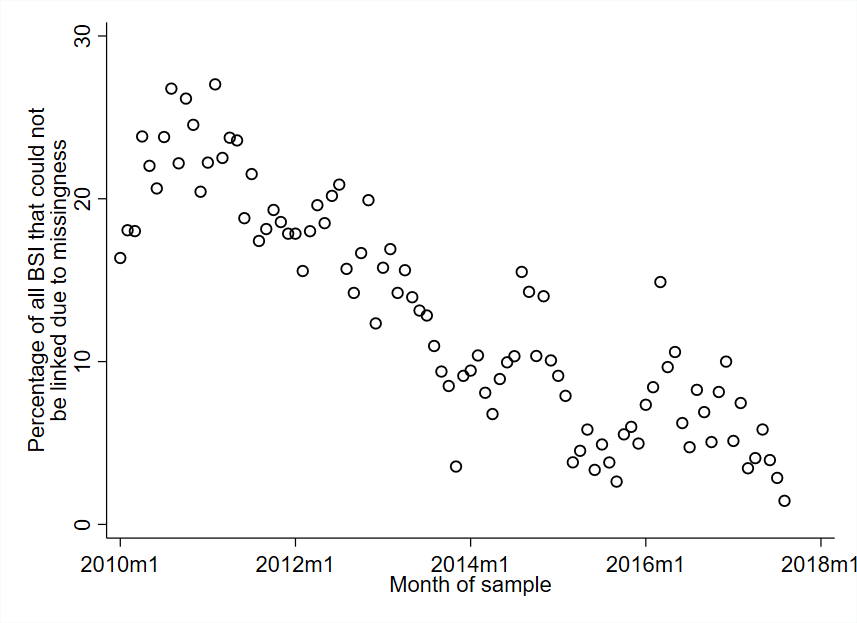

Supplement: S2 Fig — (PNG) [file pone.0226040.s005.png]
